# Peroxisome calcium uptake is dependent on ER-peroxisome membrane contact

**DOI:** 10.1007/s00018-026-06191-4

**Published:** 2026-05-12

**Authors:** Julia Kalinowski, Yelena Hartmann, Alexander Lütkemeyer, Sven Thoms

**Affiliations:** 1 Department for Biochemistry and Molecular Medicine, Medical School OWL, Bielefeld University, Bielefeld, Germany; 2 Department of Cardiovascular Diseases, German Heart Center, School of Medicine and Health, TUM University Hospital, Technical University of Munich, Munich, Germany

**Keywords:** Membrane contact site, MCS, FRET sensor, Calcium signaling, Peroxisomal disorders

## Abstract

Peroxisomes are small, highly dynamic organelles involved in a plethora of metabolic pathways. They are essential for the efficient exchange of metabolites and cellular messengers orchestrating intracellular signaling. Calcium (Ca^2+^) is one of the most prominent physiological signaling elements and regulates a wide variety of processes in cellular homeostasis and function. Recently, we showed that peroxisomes participate in cellular Ca^2+^ dynamics by taking up and releasing Ca^2+^ following store-operated calcium entry (SOCE), however, the mechanism of peroxisomal Ca^2+^ uptake and its modulators remained unknown. Using live cell imaging in combination with genetically encoded calcium indicators (GECI), we show that peroxisomal calcium dynamics are independent of PEX11β and the pore protein PXMP2. Instead, we find that the ACBD5-dependent membrane contact site between peroxisomes and the endoplasmic reticulum (ER) is necessary for efficient peroxisomal Ca^2+^ uptake. Further, we identify the ACBD5-dependent peroxisome-ER contact site as the major factor restricting peroxisome motility within the cell. Microtubules and SOCE stimulation exert smaller and independent effects on peroxisome motility. This work expands the range of known functions of the peroxisome-ER contact site.

**Supplementary Information:**

The online version contains supplementary material available at 10.1007/s00018-026-06191-4.

## Introduction

Calcium ions (Ca^2+^) are central players in many cellular processes and Ca^2+^ transients are known to regulate many signaling pathways [1–3]. Prominently, Ca^2+^ transients (short-lasting increases in intracellular Ca^2+^ concentration) are the central mechanism of excitation-contraction coupling in contractile muscle cells, while chronic dysregulation of Ca^2+^ homeostasis and signaling is detrimental to cells and implicated in multiple diseases including neurodegenerative disorders [4–6]. Major sources for Ca^2+^ transients are Ca^2+^ influx through the plasma membrane (PM) and efflux of Ca^2+^ from the endoplasmic reticulum (ER). Ca^2+^ is released from the ER either into the cytosol or to juxtaposed organelles through specialized compartments and membrane contact sites [7]. Release of Ca^2+^ from the ER triggers store-operated calcium entry (SOCE) from the extracellular space. Cytosolic Ca^2+^ is constantly pumped into the ER through the sarco/endoplasmic Ca^2+^-ATPase (SERCA) or exits the cell through the plasma membrane calcium ATPase (PMCA) [8].

Peroxisomes are small, membrane-bound organelles that contribute to a wide range of cellular metabolic pathways, most prominently lipid and ROS metabolism. The involvement of peroxisomes in cellular Ca^2+^ homeostasis has remained elusive. Plant peroxisomes contain targets of Ca^2+^ signaling, including catalases, peroxisomal protein kinases and the import and functionality of nitric oxide synthase [9–12]. However, there is currently no evidence for Ca^2+^ sensitive enzymes and pathways in mammalian peroxisomes. Additionally, peroxisomal Ca^2+^ concentration and uptake in response to cellular stimuli have long been a contentious topic with contradicting reports about the relationship of peroxisomal and cytosolic Ca^2+^. One study suggests the existence of a steep Ca^2+^ gradient between peroxisomes and the cytosol and very high Ca^2+^ peaks upon stimulation, while another reports that peroxisomal Ca^2+^ levels rise slowly and passively with cytosolic Ca^2+^ levels [13, 14]. More recently, we used a two-step stimulation protocol for monitoring Ca^2+^ dynamics based on SOCE and showed that peroxisomal Ca^2+^ levels closely follow cytosolic Ca^2+^ dynamics in response to stimuli that increase cytosolic Ca^2+^ concentration [15]. The intraperoxisomal basal Ca^2+^ concentration was determined to be approximately 600 nM, 6-fold higher than the cytosolic Ca^2+^ concentrations of around 100 nM [15].

We observed a delay in the decrease of intraperoxisomal Ca^2+^ levels after histamine stimulation, indicating that a mechanism other than free diffusion, e.g. a channel protein, might control or limit the flux of Ca^2+^ across the peroxisomal membrane [15, 16]. We therefore selected two proteins that had previously been considered as pores or channels in the peroxisome membrane as Ca^2+^ channel candidates. The yeast ortholog of PEX11β (PEX11) was shown to form moderately cation-selective pores when reconstituted in artificial membranes [17]. Additionally, mammalian Pxmp2 was identified as a major pore-forming component with weak cation selectivity in murine peroxisomal membranes in vitro [18]. More recently, both proteins were found not to be essential for the permeation of hydrogen peroxide across the peroxisomal membrane [19]. However, whether they are involved in the flux of Ca^2+^ across the peroxisomal membrane remains unknown.

Peroxisomes form membrane contact sites (MCS) with virtually all other organelles, facilitating the exchange of metabolites and enabling efficient metabolism [20–22]. For example, peroxisomes and the ER cooperate in the metabolism of lipids and peroxisome membrane expansion, a process that requires spatial proximity between the organelles [23]. The major peroxisome-ER (PO-ER) MCS consists of the tail-anchored peroxisomal membrane protein *Acyl-CoA binding domain containing 5* (ACBD5) and *vesicle-associated membrane protein-associated A* or *B* (VAPA/B) [23, 24]. This MCS has been shown to be important for peroxisome maintenance and lipid exchange [23, 25]. Mutations of ACBD5 lead to ataxia, leukodystrophy with retinal dystrophy, and an accumulation of very long-chain fatty acids (VLCFA), putatively a result of greatly reduced association of peroxisomes with the ER [26, 27]. In a cell culture model, loss of ACBD5 leads to a reduction of PO-ER contact which is marked as a substantial increase in peroxisomal motility [23]. Loss of ACBD5 also leads to a reduction in β-oxidation, which is independent of the tethering function of ACBD5 and suggests a function of ACBD5 as a lipid-binding co-factor for the peroxisomal fatty acid import mechanism [24].

On the ER surface, tail-anchored paralog VAP proteins (VAPA and VAPB) interact with a wide range of proteins in other intracellular membranes, making them the predominant mediators of ER membrane contact sites with other organelles, the cytoskeleton, and the plasma membrane. Both proteins exhibit a great overlap in functionality and interactome and knock-down of either VAPA or VAPB is not sufficient to disturb the PO-ER contact site, as one can compensate for the absence of the other [28, 25]. Notably, mutations in VAPB can cause neurodegenerative disorders, including Parkinson’s disease and familial Amyotrophic Lateral Sclerosis (ALS) Type 8 [29, 30]. Also, several neurodegenerative conditions show reduced VAP levels, likely impairing ER functions such as Ca²⁺ homeostasis [28, 6]. Furthermore, ion exchange has been documented at VAP-mediated microdomains, e.g. at mitochondria associated membranes (MAMs) and in mammalian neurons, where VAP proteins on the ER associate with ion channels (voltage-gated potassium channels Kv2.1 and Kv2.2) in the plasma membrane to form microdomains facilitating ion exchange [31, 32]. Membrane contact sites respond dynamically to cellular requirements and Ca^2+^ can modulate contact site formation [33]. However, whether any peroxisomal contact site responds to Ca^2+^ signaling remains unknown.

In this study, we investigate the mechanism by which peroxisomes take up Ca^2+^ and demonstrate that ACBD5-mediated association of peroxisomes with the ER is crucial for the efficient uptake of Ca^2+^ into peroxisomes.

## Results

### Peroxisomal Ca^2+^ uptake does not depend on PXMP2 or PEX11β

Previous investigations of peroxisomal Ca^2+^ dynamics show a significant delay in the decrease of intraperoxisomal Ca^2+^ levels after histamine stimulation, prompting the question whether a transport mechanism or channel protein in the peroxisomal membrane might limit the flux of calcium across the peroxisomal membrane [15].

**Fig. 1:**
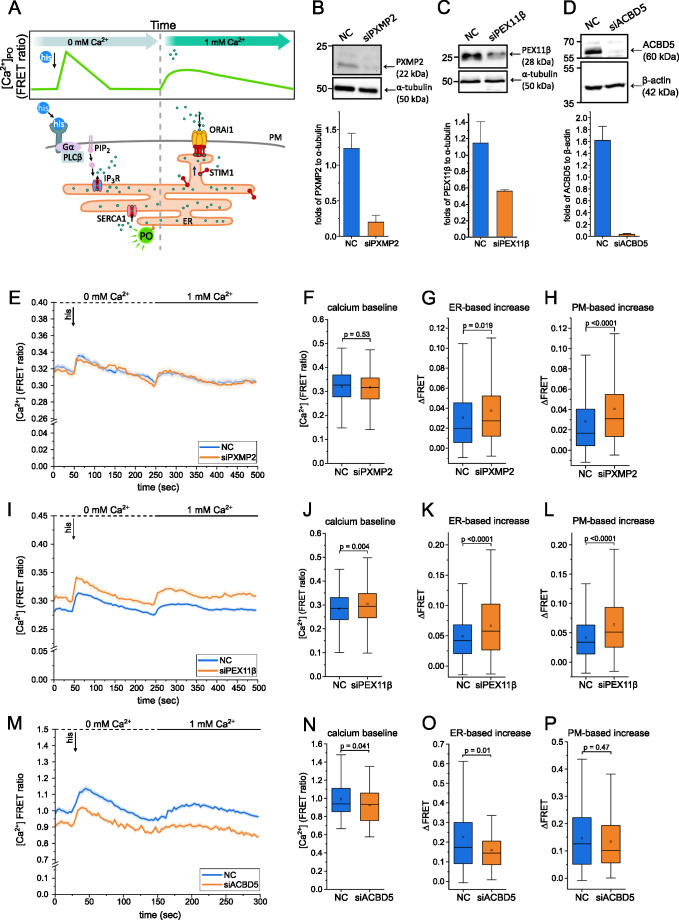
Peroxisomal Ca^2+^ dynamics are reduced in the absence of ACBD5. **A**: Experimental paradigm for analysis of peroxisomal Ca^2+^ by FRET sensor D3cpV-PTS1 in response to triggering of SOCE. **B-D**: Western Blot validation of siRNA-mediated knock-down. Representative images with quantification of band intensity of protein of interest relative to housekeeping control. Bar plot with mean ± SD. **B**: PXMP2 (*N* = 3); **C**: PEX11β (*N* = 3); **D**: ACBD5 (*N* = 4). **E**: Peroxisomal Ca^2+^ dynamics as D3cpV-PTS1 FRET ratio upon histamine-stimulation and Ca^2+^ supplementation of siPXMP2 treated cells. Mean ± SEM. *N* = 3 experiments, *n* = 221 cells (siPXMP2) and 381 cells (NC), measured over 100 imaging cycles (5 s). **F-H**: Quantification of (**E**) as baseline FRET ratio (**F**), ER-based response (**G**) and PM-based response (**H**). **I**: Peroxisomal Ca^2+^ dynamics of siPEX11β treated cells. Mean ± SEM from *N* = 6 experiments, *n* = 284 cells (siPEX11β) and 653 cells (NC), measured over 100 imaging cycles (5 s). **J-L**: Quantification of (**I**) as baseline FRET ratio (**J**), ER-based increase (**K**) and PM-based increase (**L**). **M**: Peroxisomal Ca^2+^ dynamics of siACBD5 treated cells. Mean ± SEM from *N* = 3 experiments, *n* = 149 cells (siACBD5) and 160 cells (NC), measured over 100 imaging cycles (3 s). **N-P**: Quantification of (**M**) as baseline FRET ratio (**N**), ER-based response (**O**) and PM-based response (**P**). Tukey’s box plots. P-values were calculated by two-sample t-test with Welch correction

Here, we studied peroxisomal Ca^2+^ dynamics by targeting a genetically encoded calcium indicator (GECI) directly to the peroxisomal matrix. The ratiometric D3cpV-PTS1 senses Ca^2+^ as a function of Fluorescence Resonance Energy Transfer (FRET) by conformational changes upon binding of Ca^2+^ to a calmodulin domain [15, 34]. During live-cell imaging experiments, intracellular Ca^2+^ is mobilized following a two-step stimulation protocol that recapitulates SOCE. We establish a Ca^2+^-chelating environment by incubating the cells in an EGTA-containing buffer and then add histamine as an IP_3_-generating agent leading to the efflux of Ca^2+^ from the ER via *inositol 1*,*4*,*5-triphosphate receptor* (IP_3_R). Once Ca^2+^ levels return to baseline, extracellular Ca^2+^ is supplemented which is taken up via plasma membrane channels that open in response to Ca^2+^ efflux from the ER. Peroxisomal Ca^2+^ dynamics are thus measured as the FRET ratio in response to cytosolic Ca^2+^ fluctuations during SOCE after the addition of histamine (ER-based increase) and later, the addition of external Ca^2+^ to measure the plasma membrane-based Ca^2+^ uptake (PM-based increase) (Fig. 1A). The mode of Ca^2+^ flux across the peroxisomal membrane in response to cytosolic Ca^2+^ fluctuations is unknown.

It is conceivable that channel proteins in the peroxisomal membrane might function as Ca^2+^ channels, modulating the uptake and release of Ca^2+^ by the peroxisome. PEX11β and PXMP2 have previously been investigated as peroxisomal membrane channels due to their pore-forming properties [17–19]. We selected them as candidates for potential peroxisomal Ca^2+^ channels and knocked down each factor using siRNA targeting PEX11β and PXMP2 in HeLa cells (Fig. 1B, C).

PEX11β siRNA treatment inadvertently led to some cytotoxicity which may have caused a selective enrichment of cells exhibiting stronger responses (Fig. 1I-L). When we applied the two-step protocol triggering SOCE to assess their impact on peroxisomal Ca^2+^ dynamics [15], neither of the knock-downs displayed reduced peroxisomal Ca^2+^ transients when compared to non-targeted control (NC) siRNA (Fig. 1E-L), suggesting that neither PEX11β nor PXMP2 is essential for the flux of Ca^2+^ across the peroxisomal membrane.

### The ACBD5-dependent contact site is necessary for efficient Ca^2+^ transfer into the peroxisome

Peroxisomes are known to associate with the ER, the major intracellular Ca^2+^ store which is the origin of major Ca^2+^ signaling events that can modulate organelle metabolism. Ca^2+^ plays an important role in ER-organelle contact sites: For example, mitochondrial Ca^2+^ uptake relies on the formation of high-[Ca^2+^] microdomains [35, 36] mediated by the interaction of VAPB on the ER with mitochondrial membrane protein PTPIP51 [37]. It is unlikely that peroxisomes take up Ca^2+^ from mitochondria, as knock-down of *mitochondrial calcium uniporter* (MCU) does not affect peroxisomal Ca^2+^ transients [15].

To establish the effect of peroxisomal association with the ER on peroxisomal Ca^2+^, we used siRNA to knock-down ACBD5, the predominant peroxisomal protein involved in tethering peroxisomes to the ER [24]. ACBD5 protein expression can be reliably reduced by siRNA treatment without disrupting peroxisome number and morphology (Fig. 1D, also see Fig. 4A-C). Upon knock-down of ACBD5 and thus disruption of the PO-ER tether, the baseline Ca^2+^ level (FRET ratio) is reduced (Fig. 1M, N) which might be a reflection of the slightly reduced cytosolic Ca^2+^ baseline (Fig. S1A-D) and the stimulation-dependent ER-based peroxisomal Ca^2+^ transient is decreased (Fig. 1M, O) while the PM-based increase is unchanged (Fig. 1P). This suggests that an intact membrane contact between peroxisomes and ER is necessary for efficient transfer of Ca^2+^ into the peroxisome.

### Artificial tethering of peroxisomes to the ER increases ER-based Ca^2+^ uptake

**Fig. 2:**
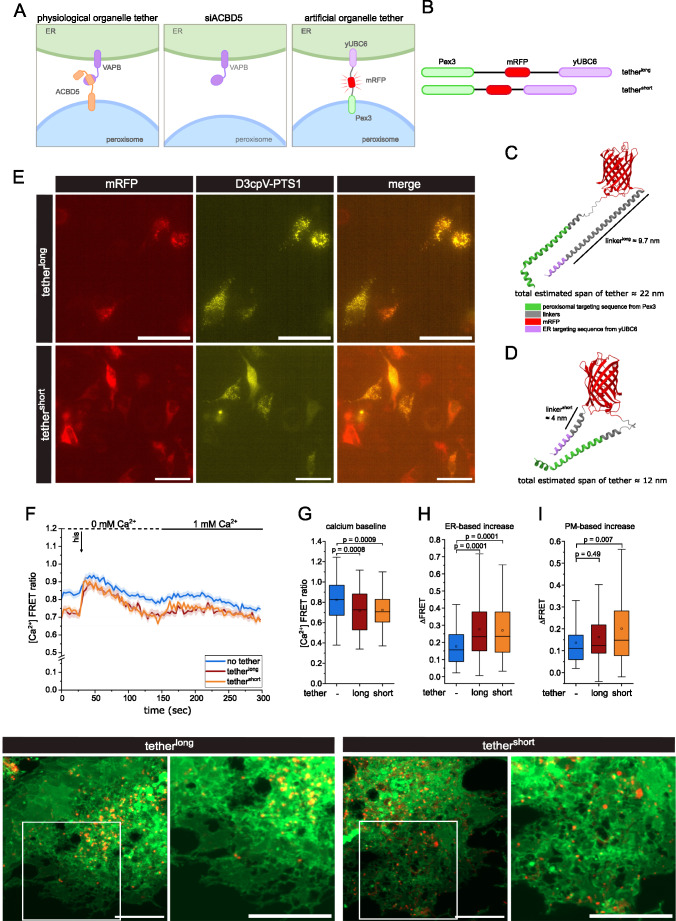
Tethering constructs force association of peroxisomes to the ER. **A**: Peroxisome-ER contact with physiological tethers, ACBD5 depletion, and expression of artificial tethering constructs. **B:** Schematic domain structure of tethers. **C**, **D**: Tertiary protein structure of tether^long^ (**C**) and tether^short^ (**D**) construct as predicted by AlphaFold2 with different domains indicated by color. Size estimations in nm of linkers and full tether construct were derived from PyMol. **E**: Images from live cell imaging of HeLa cells co-transfected with D3cpV-PTS1and tether^long^ or tether^short^. 40x magnification, scale bar 100 μm. **F**: Peroxisomal Ca^2+^ measurements of cells co-transfected with D3cpV-PTS1 and tether^long^ or tether^short^. Curves represent FRET ratios over time (mean ± SEM), measured over 100 imaging cycles (3 sec). **G-I**: Quantification of (**F**) as Ca^2+^ baseline (**G**), ER-based Ca^2+^ response (**H**) and PM-based Ca^2+^ response (**I**). Tukey’s box plots. N = 4, n = 87 (no tether), 78 (tether^long^), 100 (tether^short^). P-values calculated by one-way ANOVA with Tukey’s HSD

To further investigate the effect of peroxisome-ER association on peroxisomal Ca^2+^ dynamics, we employed artificial tether proteins (Pex3-mRFP-yUBC6) containing transmembrane domains targeting the peroxisomal membrane (Pex3) and the ER (yUBC6), respectively (Fig. 2A, B) [23]. Two tethering construct variants were used, differing in the length of the C-terminal linkers between the tethering components (Fig.2B - D). Both constructs span distances within the common estimation of MCSs distance between organelle membranes (roughly 10–30 nm) [38]. The variants will be referred to as *tether*^*long*^ and *tether*^*short*^ in the following. Both tethers contain mRFP that allows tracking of the expression and localization of the constructs. 

When we expressed the tether variants and analyzed them by live-cell imaging, we found that the tethers differ in their subcellular localization. Tether^long^ signal localized to granular structures which are retained in the perinuclear region and frequently correspond with D3cpV-PTS1 localized to peroxisomes as well as peroxisomal markers, while tether^short^ often yields a weaker, more diffuse signal in live cell imaging, especially in cells co-expressing D3cpV-PTS1 (Fig. 2E, Fig. S2B and D). The tether^short^ construct exhibited a different intracellular expression pattern compared to tether^long^. It is more often found localizing close to other cellular structures instead of a granular pattern in the perinuclear region (Fig. 2E, Fig. S2A, D). Co-expression of ER-mNeonGreen shows signal for both tether^long^ and tether^short^ localizing at the ER with a marked difference in the intracellular distribution pattern (Fig. S2G, H). This indicates that tether^long^ is more efficient in tethering peroxisomes to - or retaining them at - the perinuclear ER compared to tether^short^. 

The tether constructs were co-transfected with D3cpV-PTS1 24 h before Ca^2+^ measurements and stimulation experiments on double transfected cells were performed as previously described. Co-expression of either artificial peroxisome-ER tether in wild-type HeLa cells lowered the baseline Ca^2+^ (Fig. 2F) which partially reflects a lowered cytosolic Ca^2+^ baseline (Fig. S1E-H). The histamine-induced ER-based Ca^2+^ increase is also significantly higher in cells transfected with either tether, compared to control (Fig. 2H). The PM-based Ca^2+^ increase is only significantly increased in tether^short^ cells (Fig. 2I). Forcing association of peroxisomes to the ER therefore increases the efficiency of peroxisomal Ca^2+^ uptake while lowering baseline Ca^2+^.

### Co-transfection of artificial tether partially restores peroxisomal Ca^2+^ dynamics upon ACBD5 depletion

**Fig. 3:**
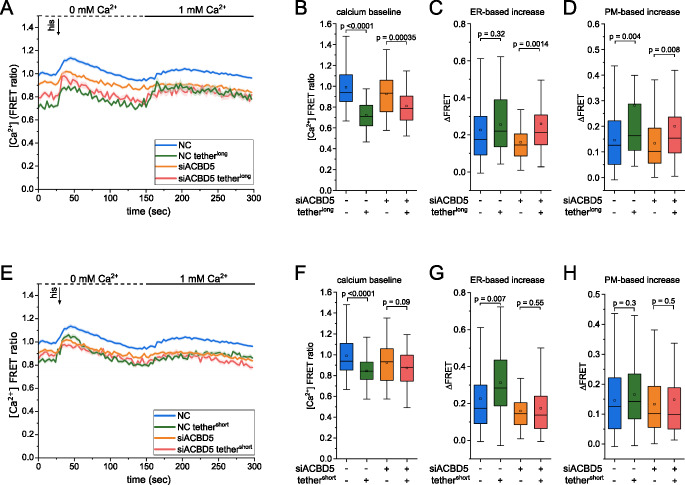
Expression of PO-ER tether restores peroxisomal Ca^2+^ uptake upon ACBD5 knock-down. A: Peroxisomal Ca^2+^ measurements of cells co-transfected with D3cpV-PTS1 and tether^long^ and siRNA targeting ACBD5. Curves represent FRET ratios over time (mean across all cells ± SEM), measured over 100 imaging cycles (3 sec). **B-D:** Quantification of (**A**) as Ca^2+^ baseline (**B**), ER-based Ca^2+^ response (**C**) and PM-based Ca^2+^ response (**D**). N = 3, n = 160 (NC), 149 (ACBD5), 61 (NC + tether^long^), 50 (ACBD5 + tether^long^). Data represented as mean ± SD. P-values calculated by two-sample t-test. **E:** Peroxisomal Ca^2+^ response in cells co-transfected with D3cpV-PTS1 and tether^short^ and ACBD5 knock-down. FRET ratios over time (mean across all cells ± SEM), measured over 100 imaging cycles (3 sec). **F-H: **Quantification of (**E**) as Ca^2+^ baseline (**F**), ER-based Ca^2+^ response (**G**) and PM-based Ca^2^^+^ response (**H**). N= 3, n = 160 (NC), 149 (ACBD5), 64 (NC + tether^short^), 59 (ACBD5 + tether^short^). Tukey’s box plots. P-values calculated by two-sample t-test with Welch correction

To study whether the enhanced association of peroxisomes and ER can compensate for the reduced uptake upon ACBD5 depletion, siRNA-treated cells were co-transfected with D3cpV-PTS1 and either tether^long^ or tether^short^. As previously established, depletion of ACBD5 leads to a drop in Ca^2+^ baseline and ER-based Ca^2+^ uptake compared to control cells (Fig. 1A). Expression of PO-ER tethering tether^long^ constructs further lowers the baseline significantly in ACBD5-depleted cells and control cells (Fig.3A, B). Upon tether^long^-expression, ER-based Ca^2+^ uptake is increased in ACBD5 siRNA-treated cells but not in control cells (Fig. 3C). PM-based Ca^2+^ uptake is increased only in control cells and - with the same trend but not significantly - in ACBD5-depleted cells (Fig. 3D). Therefore, expression of tether^long^ to force association of peroxisomes to the ER leads to increased calcium peaks in cells and rescues peroxisomal ER-based Ca^2+^ uptake after depletion of the physiological ACBD5 tethering protein while also increasing the PM-based Ca^2+^ uptake.

Expression of tether^short^ does not significantly impact Ca^2+^ dynamics in ACBD5-depleted cells as baseline Ca^2+^, ER-based Ca^2+^ uptake, and PM-based Ca^2+^ uptake remain largely unaffected (Fig.3E-H). However, in cells treated with negative control siRNA, tether^short^ has a similar effect to tether^long^ as the baseline Ca^2+^ is significantly lower and ER-based Ca^2+^ uptake is higher (Fig. 3F). Expression of tether^short^ does not ameliorate the decreased Ca^2+^ dynamics after depletion of ACBD5, possibly because it localized less to peroxisomes, but increased Ca^2+^ uptake in control cells albeit with lower baseline Ca^2+^.

Interestingly, our data indicates that the intracellular localization of peroxisomes as a result of artificial tether expression appears to influence peroxisomal Ca^2+^ uptake, especially in the absence of physiological ACBD5-VAPA/B tethers. Peroxisomes in tether^long^-expressing cells accumulate in the perinuclear region and the diminished Ca^2+^ dynamics in siACBD5 cells are reconstituted upon tether^long^ expression. In contrast, peroxisomes in tether^short^-expressing cells are distributed throughout the cell center and into the periphery and while the expression of tether^short^ still increases Ca^2+^ uptake from the ER in control cells, there is no reconstitution of Ca^2+^ uptake in ACBD5-depleted cells.

### Peroxisome motility as a readout for PO-ER contact site formation

The dynamic regulation of organelle association enables rapid adaptation to changing cellular and metabolic requirements. Phosphorylation of ACBD5 FFAT domain inhibits the interaction with VAPB and reduced PO-ER tethering [39]. Furthermore, Ca^2+^ transients can modulate contact site formation [40] and since peroxisomal Ca^2+^ dynamics are disturbed if the peroxisomal contact site protein ACBD5 is depleted, we decided to study the dynamics of the ACBD5 PO-ER contact site during Ca^2+^ stimulation more closely.

**Fig. 4:**
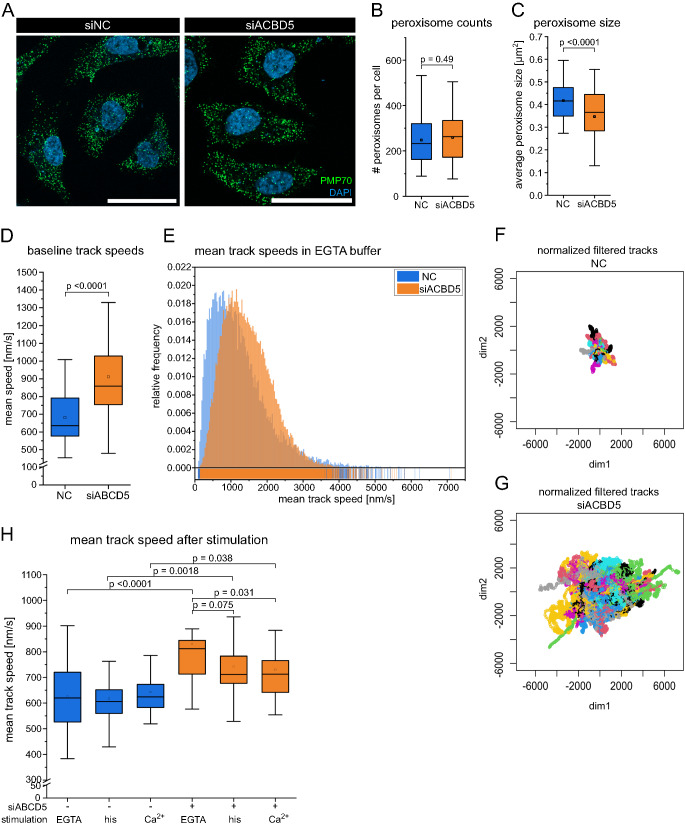
Increase in peroxisomal motility upon disruption of the PO-ER contact site and Ca^2+^-dependent decline in peroxisome motility of ACBD5-depleted cells. siRNA transfection 72 hours, HaloTag-SKL transfection 24 hours, labeling with HaloTag ligand for 1 hour before live cell recording of peroxisome motility. **A**: Representative immunofluorescence images of peroxisomes in HeLa cells treated siRNA targeting ACBD5. Cells immunostained against PMP70 (green), nuclei labeled with DAPI (blue). 63x magnification, scale bar = 50 μm. **B:** Quantification of the number peroxisomes per cell n = 95 cells (NC), n = 70 cells (siACBD5), Tukey’s box plots, p-values obtained with two-sided t-test with Welch correction. **C**: Average size of peroxisomes per cell counted in (**B**) in μm², n = 95 cells (NC), n = 70 cells (siACBD5), Tukey’s box plots, p-values obtained with two-sided t-test with Welch correction. **D**: Mean track speed in baseline conditions (DMEM with 10% FBS). Data obtained in N = 3 independent experiments, n = 27 (NC), n = 31 (siACBD5). Each cell represents an average of all (100-300) peroxisomal tracks within one cell. Tracks shorter than minimum duration of 5 seconds were excluded from analysis. **E**: Histogram of all mean track speeds of all tracks included in experiments (EGTA buffer). Exclusion of very short tracks (< 5 sec). Histogram bin size 35. **F, G**: Rose plots of normalized tracks from one representative cell treated with control siRNA (NC, **C**) and siACBD5 (**D**). **H**: Mean track speeds of cells after Ca^2+^ stimulation, filtered to include tracks with minimum 10 sec duration. N = 6, n = 22 cells (ACBD5), n = 20 cells (NC). Tukey’s box plots, p-values obtained with two-sided t-test with Welch correction (**A**) or two-way ANOVA with Bonferroni post-hoc test for repeated measurements (**E**)

As previously shown by Costello and colleagues, disruption of the ACBD5-VAPB peroxisome contact site to the ER by means of ACBD5-targeted RNA interference leads to an increase in peroxisome motility, establishing peroxisome motility as an indicator for the state of the PO-ER MCS [23]. Peroxisome motility as a read-out for MCS formation requires life cell microscopy methods with high temporal resolution due to dynamic and short nature of Ca^2+^ transients after stimulation. Depletion of ACBD5 does not affect the number of peroxisomes per cell and slightly reduces mean peroxisome size as a result of reduced membrane expansion indicating that there are no strong confounding effects on peroxisome biogenesis that are introduced by siRNA treatment (Fig. 4B, C) [23]. 

Peroxisomes employ different modes of movement, including undirected migration through the cell and relatively rare events of directed movement along microtubules, which are characterized by different speeds and directionality [41, 42]. For our purposes, we deemed the overall mean speed of peroxisomes as a suitable readout to measure PO-ER association. For live cell experiments, peroxisomes were transiently transfected with HaloTag-SKL construct and then labeled with a fluorescent HaloTag ligand for imaging. To quantify peroxisome motility, we acquired 2-minute videos with a temporal resolution of 50 ms imaging intervals and applied a tracking algorithm to extract peroxisomal movement over time. 

ACBD5-depletion (siACBD5) leads to an overall increase in peroxisome motility as indicated by an increase in the mean speed of individual peroxisomal tracks in 2-minute videos (Fig. 4D). As previously reported, we observed a higher motility of peroxisomes upon disruption of the PO-ER tether [23]. This is illustrated by an overall higher mean speed (Fig. 4D, E) and a greater range of movement (Fig. 4F, G) and confirms peroxisome motility as a suitable indicator for the disruption of the PO-ER contact site.

### Ca^2+^-dependent decline in peroxisomal motility upon ACBD5 depletion

We postulated that triggering SOCE and thus subjecting the cell to high Ca^2+^ transients might affect the degree of peroxisomes associating with the ER. If Ca^2+^ signaling led to an increase in PO-ER association, we would have expected a decrease in motility of control cells and ACBD5 siRNA cells to be unaffected. Indeed, the mean speed of peroxisomes in control cells does not change upon SOCE stimulation (Fig.4H). However, in ACBD5-depleted cells the mean peroxisome speed is in fact decreased following stimulation with histamine and Ca^2+^ supplementation (Fig. 4H). The addition of Ca^2+^ does not appear to affect PO-ER contact in control cells, since the motility of control cells does not change (Fig. 4H). On the other hand, the motility of ACBD5-depleted cells surprisingly decreases after stimulation, possibly due to other mechanisms of PO attachment to intracellular structures that are independent of ACBD5 or due to association of peroxisomes to structures other than the ER upon Ca^2+^ stimulation (Fig. 4H). 

### Nocodazole treatment has a freeing effect on peroxisomes and leads to an increase in motility independent of ACBD5

Peroxisomes not only associate with other organelles, but also with the cytoskeleton. Fast, directed transport of peroxisomes is thought to occur along microtubules, with variants of *mitochondrial Rho GTPase-1* (Miro1) localizing to the peroxisomal membrane and serving as a membrane adaptor for microtubule-dependent motor proteins kinesins and dyneins [43, 41, 20, 44]. Both, Miro1 and Miro2, are Ca^2+^-sensing proteins on the outer mitochondrial membrane and have been shown to control mitochondrial movement along microtubules, dependent on neuronal Ca^2+^ concentration [45].

We wanted to investigate the contribution of the microtubule network to the increased peroxisome motility that occurs upon disruption of the ER-association. We speculated that when association to the ER is no longer possible in the ACBD5 knock-down, peroxisomes might increase their association with microtubules, leading to fast, directed transport which would account for the increased motility that we observed [41]. In this case, disruption of the microtubule network could lead to a decrease of peroxisomal motility in siACBD5 compared to control siRNA-treated cells.

**Fig. 5:**
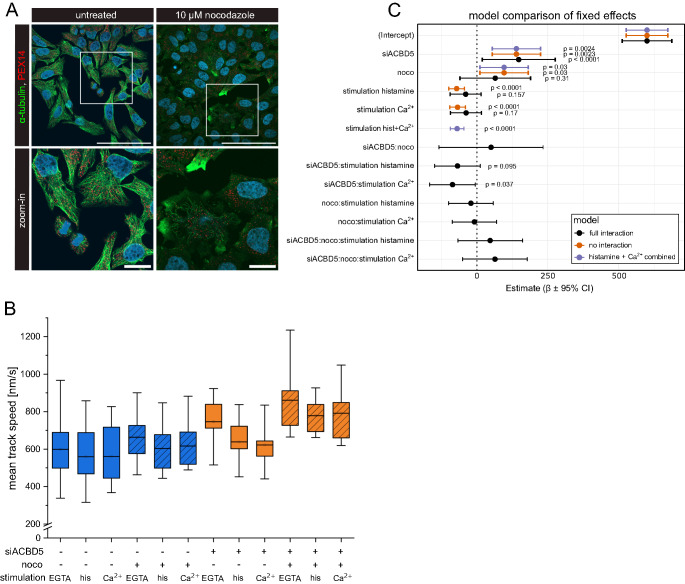
Independent effects of siACBD5, SOCE stimulation, and microtubule disruption. **A**: Nocodazole treatment disrupts the microtubule network. Immunofluorescence of HeLa cells treated with 10 µM nocodazole. Microtubules were immunostained for α-tubulin (green) and peroxisomes were immunostained for PEX14 (red). 63x magnification, scale bar 100 μm (top panels) and 20 μm (lower panels). **B**: Mean speeds of all tracked peroxisomes within a cell, filtered to include tracks with at least 10 s duration. Cells were treated with siACBD5 or scrambled control (NC), 10 µM nocodazole or DMSO, and then stimulated according to the Ca^2+^-mobilization protocol. *N* = 3 independent experiments, *n* = 9–10 cells per condition. Tukey’s box plots. **C**: Linear mixed-effects model based on (B). Representing the full interaction model (‘full interaction’, black), and the two last model reduction containing no interactions (‘no interactions’, orange), and a combination of both stimulation steps into one category (‘histamine + Ca^2+^ combined’, violet). ‘Intercept’ represents the mean speed [nm/s] of all control conditions (NC siRNA, DMSO treatment, and EGTA buffer). Estimated fixed effects (β ± 95% CI) from full and reduced mixed models. Effects of siRNA treatment, stimulation, and nocodazole were stable across model versions. 95% confidence intervals for fixed effects were calculated using the Wald method

We blocked microtubule polymerization by treatment with 10 µM nocodazole for 1 h and found disrupted microtubules in almost all cells without evidence of cytotoxicity and while preserving cell morphology (Fig. 5A). Nocodazole treatment did not affect peroxisomal Ca^2+^ dynamics (Fig. S3). We then subjected all cells to SOCE stimulation and measured peroxisome motility in EGTA-buffer after addition of histamine and Ca^2+^. Nocodazole treatment led to a notable increase in mean peroxisome speed, especially in ACBD5-depleted cells (Fig. 5B). The decrease in peroxisome motility upon SOCE stimulation is also present in siACBD5 peroxisomes after nocodazole treatment, although less pronounced.

### The motility effects of ACBD5 deletion, microtubule disruption and Ca^2+^ stimulation are separable and largely independent

 We then wanted to measure the relative contributions of ACBD5 knock-down, nocodazole treatment, and SOCE stimulation and their mutual interactions on peroxisome motility. We used a linear mixed-effects regression model to reflect the complexity of three factor interactions and the repeated measurements during stimulation [46, 47]. The regression coefficient β serves as an indicator of the effect each factor and interaction has on peroxisome motility compared to control conditions (Fig. 5C). 

We started with the full interaction model and removed nonsignificant terms stepwise using F-tests to detect significant effects of individual factors and their interactions. In the initial full model, including all two- and three-way interactions, none of the three-way interaction terms reached statistical significance (‘full interaction’, siACBD5:noco:stimulation, all *p* > 0.1). We therefore find no evidence that the effect of siACBD5:noco differs across stimulation types. However, there is a significant two-way interaction between siACBD5 and Ca^2+^, leading to reduced mean speed upon SOCE stimulation (siACBD5:stimulation Ca^2+^; β = -85.9, *p* = 0.037) which is also present as a tendency in histamine stimulation (siACBD5:stimulation histamine; β = -68.2, *p* = 0.095), reflecting the stronger effect of SOCE stimulation on peroxisome motility in siACBD5 peroxisomes compared to control we previously observed (Figs.4H and 5B).

From there, we simplified the model by removing all three-way interactions and investigating all terms containing the individual factors. By this stepwise model reduction we find that none of the two-way interaction terms involving nocodazole treatment were statistically significant (*p* = 0.58). Interactions terms involving ‘noco’ were therefore dropped from the model since nocodazole treatment does not modify other effects.

Next, we tested how the effect of siACBD5 differs across stimulation types. Interactions between stimulation with histamine or Ca^2+^ and siACBD5 do not have significant effects in the simplified model (*p* = 0.12), indicating that the influence of ACBD5 siRNA is consistent across all stimulation conditions. Furthermore, there is no significant difference in the effect of histamine and Ca^2+^-stimulation compared to control (‘no interactions’, *p* = 0.81), allowing us to summarize the stimulation levels in one factor. Therefore, the final model (‘histamine + Ca^2+^ combined’) estimates only the main effects of siRNA treatment (siACBD5 vs. NC), SOCE stimulation (histamine- and Ca^2+^-stimulation pooled vs. EGTA), and nocodazole treatment (nocodazole vs. DMSO).

Overall, stepwise model simplification did not significantly worsen the model fit (joint F-test, *p* = 0.51), justifying the simplification steps and indicating that the effects of siACBD5, nocodazole treatment, and SOCE stimulation are robust and do not substantially change when the models are restricted to exclude interactions between factors. The final model only included the main effects of the three factors siRNA treatment, SOCE stimulation, and nocodazole treatment without interactions. The ACBD5 knock-down accounts for the greatest effect on peroxisome motility and significantly increased the mean peroxisome speed (β = 139.1 ± 42.4, *p* = 0.0023), whereas SOCE stimulation reduced it (β = −69.7 ± 12.2, *p* < 0.0001). Nocodazole treatment also increases peroxisome motility (β = 95.7 ± 42.3, *p* = 0.03).

In summary, linear mixed-effects modeling revealed that the effects of siRNA treatment, nocodazole, and SOCE stimulation were additive and largely independent. The strongest determinant of peroxisome motility is the ability of peroxisome to associate to the ER, which results in an increase in peroxisome motility upon ACBD5 knock-down. Disruption of the microtubule network by nocodazole treatment also results in increased peroxisomal motility. This becomes especially evident in the absence of functional PO-ER contact, possibly due to the additional loss of microtubule interaction site for peroxisomes. Finally, mean peroxisomal speed decreases upon induction of SOCE, suggesting the existence of an unknown mechanism of Ca^2+^ -dependent attachment or limiting peroxisomal motility during Ca^2+^ signaling.

## Discussion

Peroxisomal Ca^2+^ dynamics are an elusive process. We use HeLa cells as a model to measure the response to near-physiological stimulated store-operated calcium entry, which is an important mechanism of Ca^2+^ regulation in many different tissues and cell types. SiRNA-mediated knock-down of putative candidates for peroxisomal Ca^2+^ channels, PXMP2 and PEX11β, did not negatively impact peroxisomal Ca^2+^ dynamics. The depletion of single pore-forming components of the peroxisomal membrane does not substantially affect the in- and efflux of Ca^2+^ across the peroxisomal membrane in response to stimulation. Likely, the uptake of Ca^2+^ by peroxisomes does not depend on a single membrane channel or transporter but possibly a variety of peroxisomal membrane proteins with and without known channel-forming and transporter properties creating a potential redundancy regarding the function and selectivity of the different molecular pores allows them to compensate each other’s absence [48].

Ca^2+^ plays an important role in the formation and function of membrane contact sites between the ER and other organelles, such as the plasma membrane and mitochondria. These MCS are critical for various cellular processes, including lipid exchange, Ca^2+^ signaling, and organelle communication. Peroxisomes are known to localize closely to the ER (or SR in muscle cells), the major intracellular Ca^2+^-storing compartment [49–52].

Disruption of ACBD5-mediated MCS between peroxisomes and the ER by ACBD5 knock-down lowers peroxisomal Ca^2+^ levels and reduced Ca^2+^ uptake following release from the ER upon histamine stimulation. The recent discovery that peroxisomes also form membrane contact sites with mitochondria via interaction of ACBD5 and PTPIP51 raises the question whether peroxisomes can also take up Ca^2+^ via mitochondria [53]. We previously showed that knock-down of the mitochondrial uniporter and thus disruption of mitochondrial Ca^2+^ uptake has no effect on peroxisomal Ca^2+^ dynamics, indicating that Ca^2+^ exchange between peroxisomes and mitochondria does not fundamentally contribute to peroxisomal Ca^2+^ uptake upon SOCE induction [15].

The reduced Ca^2+^ exchange between peroxisomes and ER when the MCS was disrupted could be partially reconstituted by expression of an artificial PO-ER tether construct, illustrating that the physical association of peroxisomes with the ER is necessary for efficient Ca^2^^+^ uptake by peroxisomes and other functions of ACBD5 like its contribution to lipid-transfer, are less important for Ca^2+^ exchange. Interestingly, only the tether^long^ construct was able to sufficiently rescue peroxisomal Ca^2+^ uptake after ACBD5 depletion. This was quite surprising since we expected closer membrane apposition to facilitate Ca^2+^ exchange. Therefore, we suggest that the increased ER-based Ca^2+^ uptake in tether^long^ transfected cells lacking ACBD5 might be a reflection of the differing intracellular localization of the two tethering constructs. Tether^long^ is often found to cause accumulation of peroxisomes at the perinuclear ER. This position might be advantageous in facilitating the exchange of other signaling molecules and metabolic intermediates. Different types of ER subdomains could contribute to the efficiency of peroxisome Ca^2+^ uptake. ER sheets which are more commonly found around the nucleus compared to ER tubules have been previously found to exclusively harbor ER-mito MCS that form microdomains for efficient Ca^2+^ transfer [54]. Alternatively, it has been suggested that MCSs < 10 nm may exclude bulkier membrane proteins which could apply to yet unknown channel proteins in the peroxisome or ER membrane mediating Ca^2+^ exchange between these two organelles [36]. The physiological ACBD5-VAPB contact site has been functionally defined to span distances of < 15 nm [23]. The presence of a semi-rigid spacer in tether^long^ could contribute to an optimal distance for Ca^2+^ exchange between peroxisomes and the ER.

Beyond material exchange, membrane contact sites also keep peroxisomes physically tethered in place and restrict movement, as illustrated by increased peroxisome motility when the ER contact is disrupted [23]. Therefore, organelle motility can be used as an easily accessible indirect read-out for the stability of PO-ER MCS. Other MCS have been reported to be dynamically modulated by the presence of cellular stressors, among them a PO-mitochondria contact site constituted of ACBD5 and PTPIP51 [33, 53]. Triggering of SOCE does not appear to increase ACBD5-mediated peroxisomal association with the ER. However, Ca^2+^ and Ca^2+^ signaling have a potential effect on peroxisome association with organelles or other cellular structures independently of ACBD5. We investigated the influence of microtubules on the increased peroxisome motility observed in the absence of ACBD5 as an alternative interaction partner for peroxisomes. Disruption of the microtubule network with nocodazole treatment has an additional freeing effect on peroxisomes in both control and even more pronounced siACBD5 cells. This indicates that the increase in motility seen in siACBD5 cells is not an effect of association of peroxisomes to microtubules, but presumably the result of even greater detachment from intracellular structures or greater freedom due to less spatial constraints for peroxisomes in the intracellular environment. Overall, we identify the ACBD5-mediated PO-ER MCS, microtubules, and the induction of Ca^2+^-signaling as factors modulating peroxisome motility largely independently from one another. Although all factors were shown to be acting independently from one another, the relative effects of microtubule disruption and SOCE stimulation appear more pronounced in siACBD5 cells, indicating that intact attachment to the ER in control cells may mask the effects of other factors on peroxisome motility and highlighting PO-ER contact as the main restricting factor to peroxisome motility. Other, more sensitive imaging approaches are needed to investigate the membrane contact site in more detail, for example the surface area engaged in the MCS in response to stimuli, both with regard for the high temporal dynamic of this process and the possible heterogeneity of PO-ER contact sites within one cell [54].

Taken together, this indicates that the PO-ER contact site is required for Ca^2+^ transfer to the peroxisome, and this MCS is likely a stronger constraint on the intracellular attachment of peroxisomes than microtubules.

ACBD5-VAPB interaction can be dynamically regulated by phosphorylation of the FFAT motif in ACBD5 [39]. The kinase GSK3β can phosphorylate ACBD5-FFAT, and subsequently destabilize the interaction with VAPB. Stabilizing the PO-ER contact site by favorable phosphorylation states would presumably facilitate Ca^2+^ exchange at this microdomain along with other metabolites. However, how this process is physiologically regulated by GSK3β and possibly other kinases remains largely unknown.

Another intriguing point is the protein composition of the PO-ER MCS apart from ACBD5-VAPB. ACBD5 itself is required for the efficient transfer of VLCFA to the peroxisome [24]. Whether ACBD5 could similarly support the transfer of Ca^2+^ to peroxisomes is unclear. As a tail-anchored protein, it is unlikely to form an ion channel in the peroxisomal membrane and interactions with Ca^2+^-sensitive proteins are not known. However, it is conceivable that ACBD5 may contribute to the recruitment of still unknown protein components of the PO-ER contact site similar to the protein diversity of other contact sites [22, 55, 56].

Currently, our experiments are limited to HeLa cells as they exhibit both a sufficiently good response to histamine stimulation and are morphologically compatible with live-cell imaging. However, peroxisome abundance and function may vary by tissue and cell type. Further studies will reveal peroxisomal dynamics and interactions in response to Ca^2+^ beyond the PO-ER MCS, illuminating the intricate interplay of organelles in cellular Ca^2+^ handling. In particular, investigations of the involvement of peroxisomes in electrically excitable cells in brain and muscle with their known reliance on Ca^2+^ signaling might offer intriguing insights into the role of peroxisomes in cellular Ca^2+^ homeostasis.

## Materials and methods

### Plasmids and antibodies

All plasmids were amplified using DH5α competent *E. coli* (Table 1). The plasmids encoding D3cpV-PTS1 is described in a previous publication [15]. EYFP (Clontech) and ECFP (Clontech) were used as acceptor and donor controls for the determination of bleed-through coefficients used live-cell Ca^2+^ measurements. The PO-ER tether constructs Pex3-RFP-yUBC6 long and Pex3-RFP-yUBC6 short were kindly gifted to us by Dr. Joseph Costello [23]. Both tether constructs contain the first 44 N-terminal amino acids of PEX3 targeting the peroxisomal membrane, followed by an N-terminal linker (22 aa, LELKLRILQSTVPRARDPPVAT) and mRFP1.2, then C-terminal linkers of differing length (short: 14 aa, long: 61 aa). The long linker is an extension of the short linker and contains multiple EAAAR amino acid repeats which form a semi-rigid α-helical spacer peptide. Finally, both tether constructs contain a C-terminal ER-membrane targeting sequence of yUBC6 (Fig. 2A-D). HaloTag7-SKL construct (addgene #175532) was used for labeling of peroxisomes in motility studies.

**Table 1:** Plasmids

Construct	Source
D3cpV	RRID: Addgene_36323
D3cpV-PTS1	Sargsyan et al., 2021
Pex3-RFP-yUBC6 (short)	kindly provided by Joseph Costello (University of Exeter)
Pex3-RFP-yUBC6 (long)	kindly provided by Joseph Costello (University of Exeter)
ER-mNeonGreen	RRID: Addgene_137804
HaloTag7-SKL	RRID: Addgene_175532
pECFP-C1	Clontech (TaKaRa)
pEYFP-C1	Clontech (TaKaRa)

### Cell culture and transfection

HeLa cells were cultured under standard conditions in Dulbecco’s Modified Eagle Medium (DMEM, BioWest) containing 4.5 mg/L D-Glucose, supplemented with 10% FBS and 1% Penicillin-Streptomycin, at 37 °C in 5% CO_2_ and 95% humidity atmosphere.

For live-cell imaging experiments, the cells were seeded in 8-well µ-slides (IBIDI) or 30 mm glass-bottom dishes (MatTek). For protein extraction and Western blot, the cells were seeded in a 12- or 6-well format. For cell fixation and immunofluorescence, the cells were seeded on coverslips in a 12-well format.

Transfections of plasmid DNA (Table 1) were performed with Lipofectamine™ 2000 transfection reagent (Invitrogen) 24 h before live cell imaging. For experiments requiring the co-transfection of two constructs, the overall amount of DNA was unchanged. SiRNA was transfected using Lipofectamine™ 2000 transfection reagent or Lipofectamine™ RNAiMax (Invitrogen) 72 h before experiments and knock-down was confirmed by Western Blot (Fig. 1B-D). All transfections were performed according to the manufacturer’s instructions unless otherwise indicated. The siRNA reagents used in this study include pre-designed Flexitube siRNA (Qiagen, siPEX11b: SI04152673 and SI04373201, siPXMP2: SI03183243 and SI04225879) and Silencer™ Select pre-designed siRNA targeting ACBD5 (4392420, Life Technologies) and AllStars Negative Control siRNA (1027281, Qiagen). All measurements were performed 72 h after siRNA transfection and the knock-down efficiency was confirmed by Western Blot.

For disruption of the microtubule network, cells were treated with 10 µM nocodazole (Sigma-Aldrich) in standard growth medium as a control, a cohort of cells was treated with an equivalent volume of DMSO (Roth) in growth medium (1:10,000 dilution of DMSO (14.07 M) stock solution, 1.41 mM final concetration on cells) lower concentrations and longer incubation times were not sufficient to disrupt the microtubule network as efficiently (Fig. S3I).

The old medium was aspirated and the nocodazole working solution was added to the cells. The cells were then incubated for 1 h at 37 °C in 5% CO_2_ and 95% humidity atmosphere prior to live cell experiments or fixation.

### SDS-PAGE and western blot

For SDS-PAGE and western Blot, cells were scraped in cold PBS and the lysed in RIPA buffer (25 mM Tris-HCl, pH 8.0, 150 mM NaCl, 1% NP-40, 1% Na-deoxycholate, 2% SDS), DNA was removed from the sample either by DNaseI digestion or sonication. The protein content was determined by BCA Protein Assay (Pierce). Laemmli buffer (40 mM Tris-HCl, pH 6.8, 2% SDS, 0.025% bromophenol blue, 150 mM DTT, 5% glycerine) was the added to the sample and 30 µg of protein were loaded on an SDS-Gel (15% acrylamide separation gel for PEX11β and PXMP2, 10% acrylamide for ACBD5). The proteins were blotted on a 0.45-micron nitrocellulose membrane (Thermo Scientific) with a semi-dry blotting device (Trans-Blot Turbo, BioRad) in transfer buffer with EtOH (25 mM Tris, 150 mM glycine, 0.05% SDS, 10% ethanol, pH 8.5). After rinsing of the membrane with TBS-T (20 mM Tris base, 150 mM NaCl, 0.1% Tween-20, pH 7.6) and blocking (5% milk powder in TBS-T), proteins of interest were detected by incubating with the primary antibody (diluted in 5% milk powder in TBS-T) for 1 h at room temperature or overnight at 4 °C, and incubation with HRP-coupled secondary antibody for 1 h at RT. The membrane was covered with ECL reagent (Roche) and imaged with the ChemoStar Touch ECL & Fluorescence imager (Intas) Area and percent intensity were calculated with ImageJ (RRID: SCR_002285) [57].

### Immunofluorescence and microscopy

For immunofluorescence, cells were washed with PBS (137 mM NaCl, 2.7 mM KCl, 10 mM Na_2_HPO_4_, 1.8 mM KH_2_PO_4_) and then fixed with 4% PFA for 10 min at room temperature. The cells were then permeabilized with 0.5% Triton-X100 (Sigma-Aldrich) for 5 min at room temperature. Unspecific epitopes were blocked with 5% BSA (Roth) for 30 min at room temperature. Antibodies were diluted in PBS with 1% BSA as detailed in Table 2 and the primary antibody dilution was incubated with the cells over night at 4 °C. Secondary antibody staining was performed for 1 h at room temperature before mounting the cells on specimen slides for imaging. Imaging of fluorescently labeled cells was done with a LSM900 Laser Scanning microscope with ZEN software (Carl Zeiss AG) using a 63x oil-immersion objective. For live cell imaging of cells expressing ER-mNeonGreen with tether constructs was performed on a LSM900 Laser scanning microscope with Airyscan processing. Full Z-stacks of the cells (5–15 slices) were acquired and transformed into a maximum intensity orthogonal projection.

**Table 2:** Antibodies

Target	Supplier	Dilution
PXMP2 (rabbit polyclonal)	Proteintech Cat# 24801-1-AP, RRID: AB_2879733	1:250 (WB)
PEX11B (rabbit polyclonal)	Abcam [EPR12183] Cat# ab181066	1:1000 (WB)
ACBD5 (rabbit polyclonal)	Proteintech Cat# 21080-1-AP, RRID: AB_2878808	1:50000 (WB)
β-actin (rabbit monoclonal, SP124)	Invitrogen Cat# MA5-16410	1:1000 (WB)
α-tubulin (mouse monoclonal)	CellSignaling Cat# 3501	1:500 (IF), 1:1000 (WB)
Pex14 (rabbit polyclonal)	Proteintech Cat# 10594-1-AP, RRID: AB_2252194	1:500 (IF)
PMP70 (mouse monoclonal, 70 − 18)	Sigma-Aldrich Cat# SAB4200181, RRID: AB_10639362	1:200 (IF)
Catalase (rabbit polyclonal)	AOXRE Cat# 24,316	1:500 (IF)
ACAA1 (rabbit polyclonal)	Proteintech Cat# 12319-2-AP, RRID: AB_2289045	1:500 (IF)
α-rabbit-HRP IgG (goat polyclonal)	Jackson Immuno Research Cat# 111-035-003)	1:10000 (WB)
α-mouse-HRP IgG (goat polyclonal)	Jackson Immuno Research Cat# 715-035-150)	1:10000 (WB)
Alexa Fluor488 goat anti rabbit	Invitrogen Cat# A11008	1:1000 (IF)
Alexa Fluor 488 goat anti mouse	Invitrogen Cat# A32723	1:1000 (IF)
Alexa Fluor 647 goat anti rabbit	Invitrogen Cat# A21244	1:1000 (IF)
Alexa Fluor 647 goat anti mouse	Invitrogen Cat# A32728TR	1:1000 (IF)

### Live cell imaging for Ca^2+^ measurements

The imaging protocol with genetically encoded calcium indicators generally follows established guidelines [58]. The specific process and buffer recipes for imaging peroxisomal Ca^2+^ transients with D3cpV-PTS1 following induction of SOCE has been detailed elsewhere [59]. The bleed-through coefficients for the imaging setup and acquisition settings used for downstream data analysis were determined experimentally using single-fluorophore constructs for CFP (Clontech) and YFP (Clontech) according to the instructions given by Palmer & Tsien (2006) (Table 1) [58]. The introduction of mRFP did not alter the bleed-through coefficients after optimization of illumination settings and could therefore be neglected. All buffers and stimulants were prepared as 3x buffers, so that final concentrations of stimulants amount to 100 µM histamine and 1 mM Ca^2+^, respectively.

We used the DMi8 imager (Leica Microsystems) equipped with a Leica K8 camera, a CYR71010 filter cube, environmental control (Okolab) and a 40x objective. For excitation LEDs for 440 nm (CFP), 510 nm (cpVenus) and 575 nm (mRFP) were used with the bandpass emission filters 460/80 nm, 535/70 nm and 642/80 nm.

For experiments, all buffers were warmed to 37 °C and the microscope imaging stage was heated to 37 °C and with 5% CO_2_ atmosphere. Cells transfected with D3cpV or D3cpV-PTS1 were washed with PBS to remove residual medium, and then washed again in Ca^2+^-free standard buffer (145 mM NaCl, 4 mM KCl, 10 mM HEPES, 10 mM glucose, 2 mM MgCl_2_, and 1 mM EGTA, pH 7.4) at 37 °C). The cells were then incubated for 5–10 min in Ca^2+^-free standard buffer for equilibration prior to imaging on the pre-warmed imaging stage. To measure Ca^2+^ dynamics, the images taken every 3 to 5 s with a total of 100 imaging cycles. Ca^2+^-depletion from ER stores is achieved by adding 50% of original volume of standard buffer with histamine (standard buffer without EGTA, containing 300 µM histamine, final concentration on cells is 100 µM) after cycle 10, standard buffer with Ca^2+^ (standard buffer that contains 3 mM CaCl_2_ instead of EGTA, final Ca^2+^ on cells is 1 mM) is added after cycle 50 to enable Ca^2+^ uptake through the plasma membrane. Image acquisition was performed with LAS-X Software (Leica Microsystems), marking of regions of interest and intensity measurements were performed with ImageJ image processing software [57].

After image acquisition, individual cells were marked as regions of interest and analyzed separately. The FRET to donor ratio as a readout for calcium was calculated correcting for background and bleed-through (BT):

$$\:\frac{\mathrm{FRET}}{\mathrm{donor}}\:=\:\frac{\left(\mathrm{FRET}-\mathrm{background}\right)-\left[\left(\mathrm{CFP}-\mathrm{background}\right)\:\times\:\:\mathrm{BT}\right]-\lbrack\left(\mathrm{YFP}-\mathrm{background}\right)\:\times\:\mathrm{BT}\rbrack}{\left(\mathrm{CFP}-\mathrm{background}\right)-\lbrack\left(\mathrm{YFP}-\mathrm{background}\right)\:\times\:\mathrm{BT}\rbrack}$$

This process was automated using a custom R studio script (R Version 4.5.1). Then, the metrics of calcium baseline (mean FRET ratio of imaging cycles 1–9), the ER-based calcium increase (ΔFRET ratio of calcium baseline and maximum FRET ratio in imaging cycles 9–25), and PM-based calcium increase (ΔFRET ratio of mean FRET ration in cycles 47–49 and maximum FRET ratio in imaging cycles 49-70) were calculated.

In experiments with double transfection of artificial tether and D3cpV-PTS1, all cells with peroxisomal D3cpV-PTS1 were included in the initial intensity measurements. To identify cells expressing the tether constructs, auto-annotation and -sorting of cell values based on mRFP signal intensity was included in the data processing pipeline. Threshold values for mRFP-expression were chosen based on a pilot data and subsequent experimental data was categorized by background-corrected mean gray value into mRFP-negative (‘neg’, < 0.5), and mRFP-positive (‘pos’, > 0.5).

### Peroxisome motility measurements

For live cell experiments, HeLa cells were transfected with HaloTag7-SKL 24 h before experiments. On the day of the experiment the cells were incubated with JF657 HaloTag ligand (a kind gift from Dr. Luke Lavis, Janelia Research Campus) diluted in phenol red-free standard growth medium (Gibco) for 1 h at 37 °C with 5% CO_2_ and 95% humidity. The cells were then washed three times with phenol red-free standard growth medium. Other buffers were added to the cells according to the experimental requirements.

For experiments with a greater temporal and spatial resolution, we used the super-resolution ONI Nanoimager which allows simultaneous acquisition of two channels and, concomitantly, very high temporal resolution with one image taken every 50 ms.

Peroxisome tracking was performed with the built-in tracking algorithm (ONI, settings: max gap 0, maximum distance 0.5 μm, exclusion radius 0.03 μm) and position data was exported. Further analysis of tracking data was performed in a custom R Studio (R Version 4.5.1) analysis pipeline using the CellTrackR package [60]. This enabled data visualization, filtering of very short tracks (less than 5–10 s or 100–200 steps), determination of mean track speeds as well as the determination of instantaneous velocity (or edge speed) of peroxisomes throughout the video.

### Peroxisome counting

For peroxisome quantification, fixed and immunostained HeLa cells were imaged on the LSM900 confocal microscope (Zeiss). Cells were analyzed in ImageJ, by applying a simple threshold (system default) and watershed to isolate the peroxisomes, and marking individual cells as regions of interest. Peroxisome counts and average size for each cell were generated with the built-in particle analyzer.

### AlphaFold modelling

To approximate the 3D structure of the artificial tether constructs, we used AlphaFold2 to predict the structure and color the domains and linkers [61]. The sizes of linkers were approximated in PyMol (1 Å = 0.1 nm) [62]. For size estimation of the entire construct membrane targeting domains and linkers were manually moved into a position of maximum extension (not shown) and the distance from first to last amino acid residue measured.

### Statistical analyses

If not indicated otherwise, statistical analyses were performed with Origin Labs Software (Origin 2023b, RRID: SCR_014212) and statistical tests performed are listed in figure legend.

Peroxisome mean speeds under the influence of three factors (siRNA, nocodazole, SOCE stimulation steps) was analyzed using linear mixed-effects models implemented in the lme4 package in R [46]. Degrees of freedom and p-values for fixed effects were estimated using Satterthwaite’s approximation [47]. The dependent variable is the mean speed [nm/s]. Fixed factors include siRNA treatment (siABD5 vs. NC), nocodazole treatment (noco), and stimulation condition (EGTA, histamine, Ca^2+^), and their interactions. A random intercept for ‘Subject’ was included to account for repeated measurements within samples. Model simplifications followed a stepwise backward procedure based on F-tests of fixed effects (lmerTest::contest) and comparison of nested models. 

## Supplementary Information

Below is the link to the electronic supplementary material.

Supplementary Material 1 (DOCX 4.34 MB)

## Data Availability

Raw data of FRET measurements are available from the corresponding author on reasonable request.
