# Supplementary material for: Peroxisome calcium uptake is dependent on ER-peroxisome membrane contact
Source: Cell Mol Life Sci. 2026 May 12;83(1):207. doi: 10.1007/s00018-026-06191-4 (PMC13172193; doi:10.1007/s00018-026-06191-4)
Supplement: Supplementary file 1 — Supplementary Material 1 (DOCX 4.34 MB) [file 18_2026_6191_MOESM1_ESM.docx]

**Peroxisome calcium uptake is dependent on ER-peroxisome membrane contact**

Julia Kalinowski^1^, Yelena Hartmann^1,2^, Alexander Lütkemeyer^1^, Sven Thoms^1,*^

^1^Department for Biochemistry and Molecular Medicine, Medical School OWL, Bielefeld University, Bielefeld, Germany

^2^ Present Address: Department of Cardiovascular Diseases, German Heart Center Munich, Germany

**SUPPLEMENTARY FIGURES**
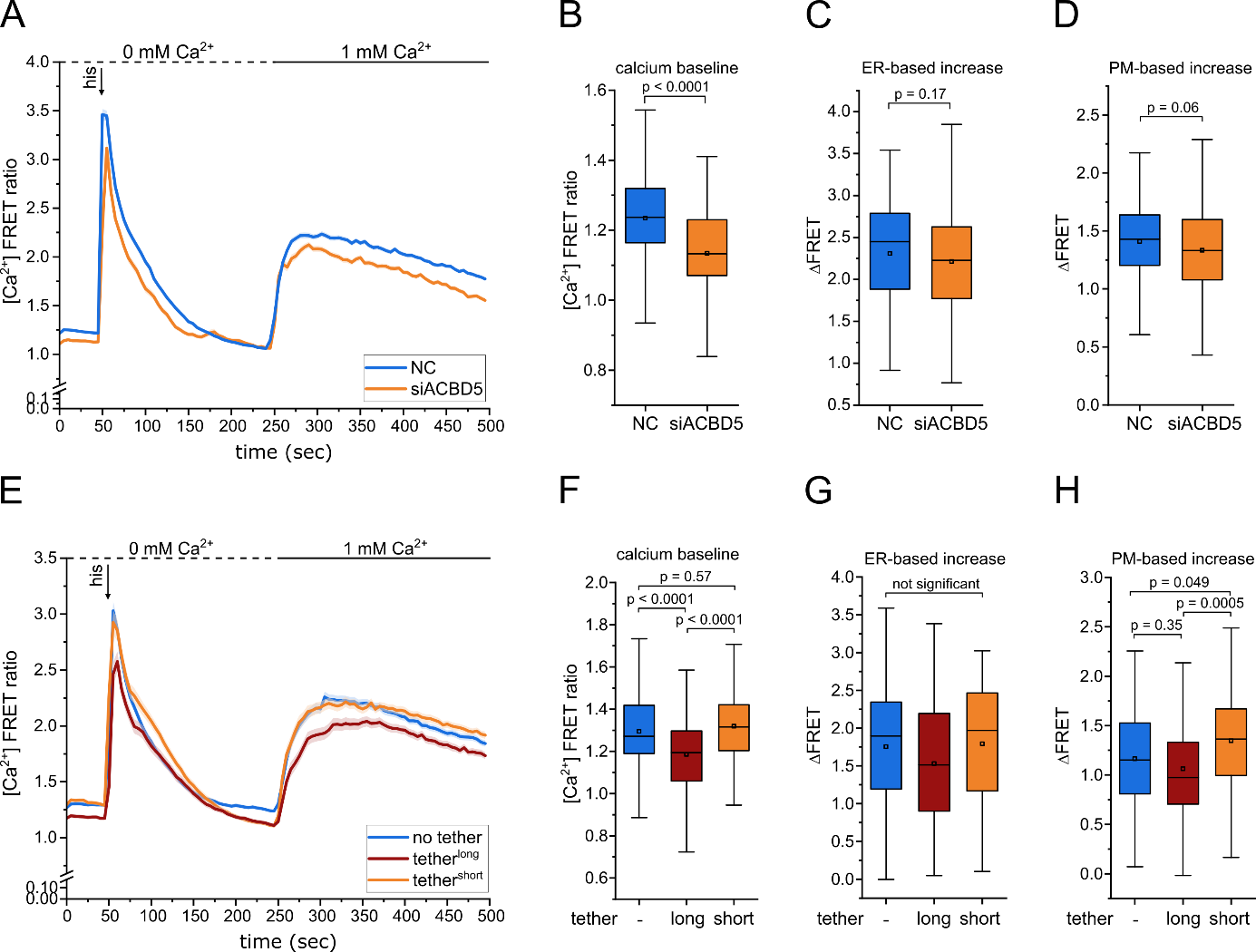


**Fig. S1.** Cytosolic calcium dynamics in HeLa cells transfected with siACBD5 or artificial tether constructs. **A:** Cytosolic Ca^2+^ measurements as FRET ratios of cell transfected with D3cpV. Curve represent mean ± SEM of N = 3 experiments, n = 176 cells (siNC) and n = 158 (siACBD5). **B, C, D**: Quantification data in A of Ca^2+^ baseline (B), ER-based Ca^2+^ increase (C) and PM-based Ca^2+^ increase (D). Tukey’s box plots, p-values were calculated by two-sample t-test with Welch correction. **E:** Cytosolic Ca^2+^ measurements as FRET ratios of cell transfected with D3cpV. Curve represent mean ± SEM of N = 3 experiments, n = 109 cells (no tether), n = 108 (tether^long^) and n = 105 (tether^short^). **F, G, H**: Quantification data in E of Ca^2+^ baseline (F), ER-based Ca^2+^ increase (G) and PM-based Ca^2+^ increase (H). Tukey’s box plots, p-values calculated by one-way ANOVA with Tukey’s HSD.

**
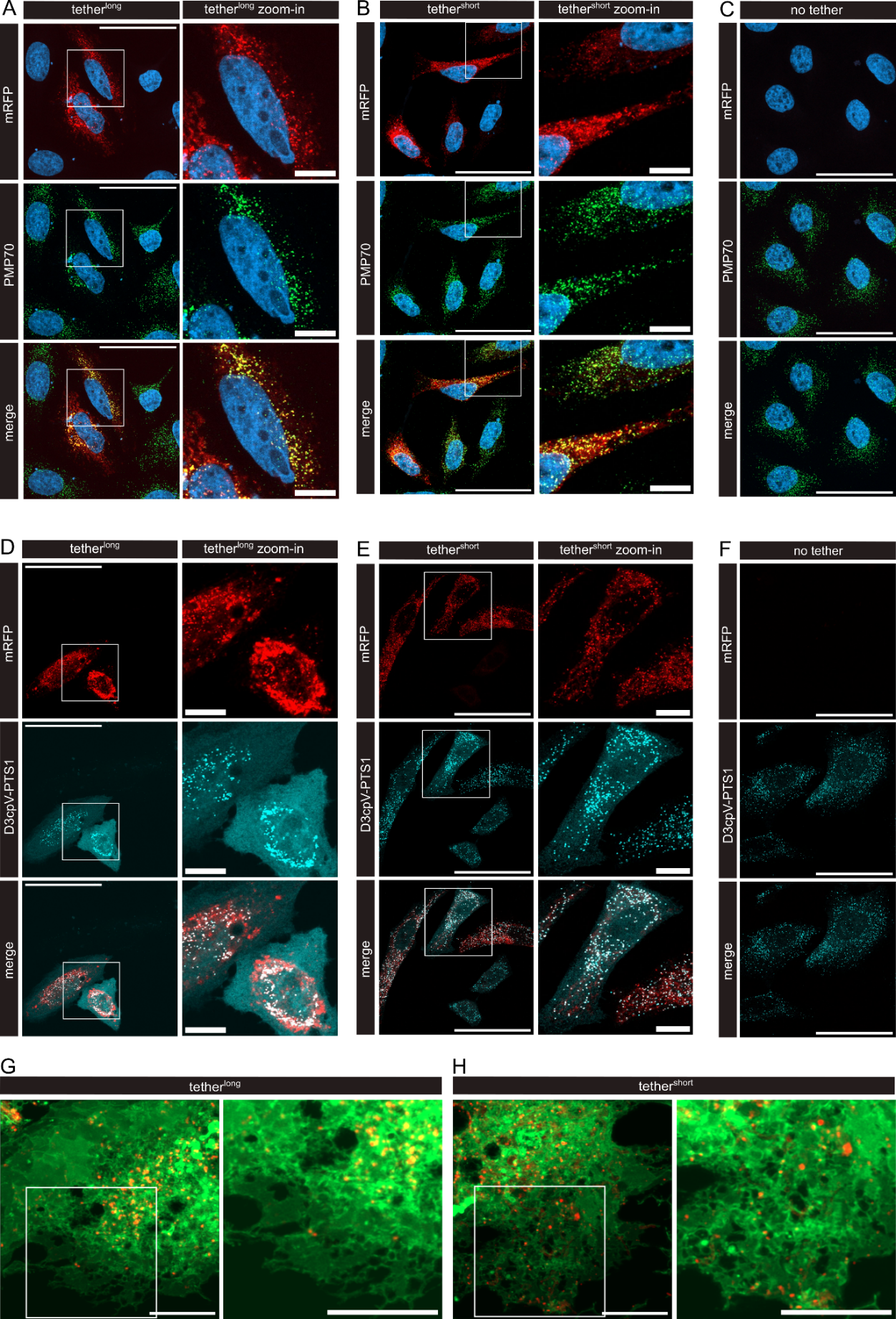
**

**Fig. S2.** Immunofluorescence of artificial tether constructs. HeLa cells fixed with 4% PFA and immunostaining against peroxisomal marker PMP70 and direct fluorescence. **A:** Tether^long^ transfected cells. **B:** Tether^short^ transfected cells. **C:** Untransfected cells. **D-F**: Expression of peroxisomal Ca^2+^ sensor D3cpV-PTS1 together with **D:** tether^long^, **E:** tether^short^. **F:** No tether co-transfected. 63x magnification, scale bar 50 µm and 10 µm (zoom-in). **G, H:** Airyscan-processed live cell images of HeLa cells expressing ER-mNeonGreen and tether^long^ (G) or tether^short^ (H). Maximum intensity projection of Z-stacks (5-13 slices) with 63x magnification with 3x zoom. Scale bar = 10 µm.

**
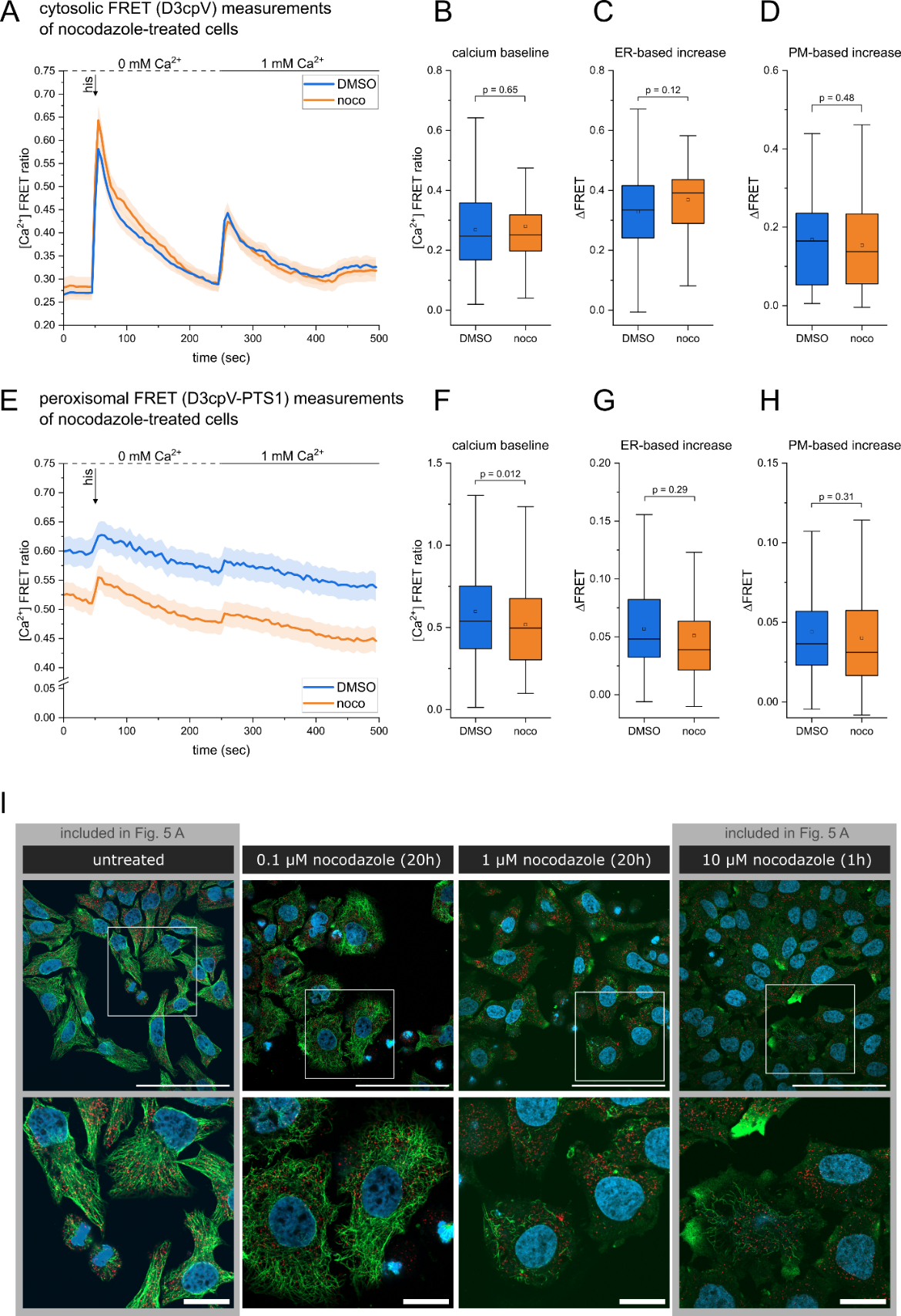
**

**Fig. S3.** Cytosolic and peroxisomal calcium dynamics in nocodazole-treated cells. **A:** Cytosolic Ca^2+^ measurements as FRET ratios of cell transfected with D3cpV. Curve represent mean ± SEM of n = 90 cells (DMSO) and n = 59 (noco). **B, C, D**: Quantification data in A of Ca^2+^ baseline (B), ER-based Ca^2+^ increase (C) and PM-based Ca^2+^ increase (D). P-values calculated by two-sample t-test, data represented as mean ± SD. **E:** Peroxisomal Ca^2+^ measurements as FRET ratios of cell transfected with D3cpV-PTS1. Curve represent mean ± SEM of n = 135 cells (DMSO) and n = 176 (noco). **F, G, H:** Quantification data in E of Ca^2+^ baseline (F), ER-based Ca^2+^ increase (G) and PM-based Ca^2+^ increase (H). Tukey’s box plots. P-values calculated by two-sample t-test. **I:** Extended data for Fig. 5 A. Immunofluorescence of the microtubule network in HeLa cells treated with different concentrations of nocodazole for different periods of time. Microtubules were immunostained for α-tubulin (green) and peroxisomes were immunostained for PEX14 (red). 63x magnification, scale bar 100 µm (top panels) and 20 µm (lower panels).
